# The relationship between gender, psychosocial factors, pain, health literacy and health-related quality of life in parents of Norwegian adolescents one year into the COVID-19 pandemic

**DOI:** 10.1186/s12889-024-18525-7

**Published:** 2024-04-08

**Authors:** Gudrun Rohde, Sølvi Helseth, Milada Hagen, Hilde Timenes Mikkelsen, Siv Skarstein, Kristin Haraldstad

**Affiliations:** 1 Department of Health and Nursing, Faculty of Health and Sport Sciences, University of Agder, Postbox 422, 4604 Kristiansand, Norway; 2 Department of Clinical Research, Sorlandet Hospital, Kristiansand, Norway; 3 Department of Nursing and Health Promotion, Faculty of Health Sciences, Oslo Metropolitan University, Oslo, Norway

**Keywords:** Health-related quality of life, Parents, Stress, Pain, Health literacy, self-efficacy, self-esteem, Loneliness

## Abstract

**Background:**

Stress impacts healthy behaviours and may influence life and health-related quality of life (HRQOL). A stressful event occurred when the COVID-19 pandemic hit in March 2020. The present study aims to explore possible gender differences in stress, psychosocial factors (self-efficacy, self-esteem, loneliness), pain, HL, and HRQOL in parents of adolescents one year into the COVID-19 pandemic, and to explore possible associations between gender, demographic and psychosocial factors, pain, HL, and HRQOL.

**Methods:**

Parents of adolescents aged 16–17 took part in the study from January to February 2021, when the COVID-19 pandemic was ongoing. Data on socio-demographics, stress, self-efficacy, self-esteem, pain, HL, loneliness, and HRQOL were collected. HRQOL was assessed using RAND-36.

**Results:**

Among the 320 parents from the general population, the mean age was 47.6 (standard deviation (SD) = 4.6) years, 81% were mothers, 79% were married or cohabiting, 81% had a university degree, and the majority worked full time (78%) or part time (13%). The average pain score was low, 0.48 (95% CI [0.43–0.54]). However, 50% of the parents reported persistent pain and more mothers reported persistent pain compared to fathers (53% vs. 37%). The parents’ mean (SD) score for RAND-36 was 52.1 (95% CI [51.2–53.0]) for the physical component summary (PCS) score and 51.0 (95% CI [50.0–52.1]) for the mental component summary (MCS) score. Mothers reported significantly lower scores for all the eight RAND-36 domains and the PCS and MCS scores. Adjusting for gender, age, living condition, education, pain, HL, self-efficacy and loneliness, we revealed no associations between stress and RAND-36-PCS. University education of four years or more was positively associated (B = 3.29, 95% CI: [0.78–5.80]) with RAND-36-PCS, while persistent pain was negatively associated (B = -7.13, CI: [-9.20– -5.06]). We identified a strong negative association between RAND-36-MCS and stress (B = -43.11, CI: [-48.83– -37.38]) and a positive association with older age (B = 0.21, CI: [ 0.04, 0.39)].

**Conclusion:**

One year into the COVID-19 pandemic, we identified a strong negative association between stress and mental HRQOL, while pain was strongly negatively associated with physical HRQOL.

## Introduction

Stress impacts healthy behaviours and may influence various aspects of life and health-related quality of life (HRQOL) [1]. According to Lazarus and Folkman, stress is a relationship between the person and an environment that the person appraises as taxing or exceeding their resources and endangering their well-being [2]. HRQOL is defined as a multidimensional concept that is used to assess subjective well-being in terms of physical, psychological, social, and spiritual aspects of life [3], and the multidimensionality of HRQOL can provide valuable information about the impact of stress and other psychosocial issues on various aspects of life [1]. Previous studies have identified associations between high levels of stress and low HRQOL [4–6].

A stressful situation occurred when the COVID-19 pandemic hit in March 2020. Recent studies have shown that the early stages of the COVID-19 pandemic were associated with increased economic and social impact, stress, loneliness, and depressive and anxiety symptoms [7–10]. This might be especially prominent for adults in charge of children and adolescents. Loneliness in this context is defined as a negative feeling experienced when there is a discrepancy between desired interpersonal relationships and the relationships an individual perceives they currently have [11]. Factors like stress, loneliness, anxiety, economic worries, and resilience factors, like general self-efficacy and self-esteem, might all influence HRQOL during a crisis like the COVID-19 pandemic. However, studies of the impact of the pandemic on mental health, loneliness, and well-being in the general population have also been quite heterogeneous, and a substantial group was found to have been either largely unaffected or even to have done better during the pandemic period [12–15]. A longitudinal study has shown signs of resilience in the general population, with a surprising ability to adapt [7].

Determinants of stress include the individuals’ ability to manage their thoughts and emotions [16], and parents’ thoughts and cognition may be essential in terms of the subjective perception of stress. According to Albert Bandura, self-efficacy might be considered a resilience factor for HRQOL [17, 18]. Previous studies have indicated that general self-efficacy (GSE) positively impacts HRQOL by reducing stress, increasing HRQOL in adult patients [19, 20]. Furthermore, studies have reported stress symptoms to be associated with a low HRQOL [21–23]. In data from the current cohort, we have previously identified associations between stress, GSE, pain, and HRQOL in adolescents and their parents before and during the COVID-19 pandemic [5, 6, 24–27].

COVID-19-related advice and information must be understood and acted upon for protective strategies against COVID-19 to be successful. Health literacy (HL) has been emphasized as crucial in dealing with and managing the pandemic [28, 29]. HL is a skill-based process that can be used to identify and transform health-related information into knowledge and action, making it vital for a person’s ability to navigate the health-care system and manage health [28]. Previous studies have shown that HL is associated with HRQOL in adolescents and adults before and during the pandemic [26, 29–33].

To be a parent and role model for an adolescent may be enriching, but also a stressful and demanding period in adult life. Health behaviour, resilient factors and patterns follow generations, like pain and pain coping and health behaviours related to the pandemic. Previous studies have revealed that parents’ pain and pain-coping patterns can be adopted by their adolescents, thereby influencing the HRQOL of both adults and adolescents [34, 35]. Pain in this context is defined as an unpleasant sensory and emotional experience associated with, or resembling that associated with, actual or potential tissue damage and including both physical and psychological pain [36].

In the transitional phase from childhood to adulthood, adolescents desire for independence and greater autonomy, their brain develops, pressure to conform to peers and the exploration of sexual identity become dominant, while rapid physical development is ongoing [37, 38]. In general, support from parents is important in this phase [39], however it might be considered vital during stressful periods like the COVID-19 pandemic. The supportive role as a parent and various psychosocial factors (stress, self-efficacy, self-esteem, loneliness), might have influenced how parents reported their HRQOL [40]. The present study aims to explore possible gender differences in stress, psychosocial factors (self-efficacy, self-esteem, loneliness), pain, HL, and HRQOL in parents of adolescents one year into the COVID-19 pandemic, and to explore possible associations between gender, demographic and psychosocial factors, pain, HL, and HRQOL.

## Methods

### Study sample

This study was part of the “Start Young—Quality of Life and Pain in Generations” study [14], a Norwegian four-year prospective study of adolescents and their parents in a school-based sample in the general population in the southern part of Norway. Detailed descriptions of this study have been published [5, 6, 24–27]. Here, we used data collected from the parents at the follow-up when the adolescents were 16–17 years old (in their first year of upper secondary school), from January to February 2021, i.e., during the COVID-19 pandemic. In total, 320 parents completed the survey and were included in the analysis.

The data were collected using a web-based questionnaire, and we used a safe data server to store the collected data [37]. All study procedures were approved by the Norwegian Centre for Research Data (Ref: 60,981).

### Instruments

#### Demographic variables

The first part of the questionnaire included self-reported data on demographic variables such as gender, age, marital status, education, household income, employment status, absence from work, and region [41].

#### HRQOL, self-efficacy, self-esteem, loneliness, stress, pain and health literacy

*HRQOL* was measured using the RAND-36 generic questionnaire. RAND-36 includes eight domains: general health, bodily pain, physical function, role limitations (physical), mental health, vitality, social function, and role limitations (emotional). These domains can be combined into two summary scales that reflect physical (physical component summary (PCS)) and mental (mental component summary (MCS)) health. The RAND-36 scales were scored according to recommended scoring procedures, and each scale was expressed using values from 0 to 100, with 100 representing excellent health [42–45]. The Cronbach’s α values in this study were satisfactory and ranged from 0.83 (general health) to 0.93 (physical function).

*Self-efficacy* was measured using the GSE scale [46, 47]. GSE consists of 10 items with a four-point scale from 1 (completely wrong) to 4 (completely right). Scores on each item were summed and divided by 10 into a GSE score in the 1–4 range. Higher scores indicate higher GSE levels. The questionnaire is valid and reliable [47]. Cronbach’s α in this study was 0.89.

*Self-esteem* was measured using a four-item short version of the Rosenberg Self-Esteem Scale (RSES) [48]. RSES consists of four self-perception statements rated on a 4-point Likert scale ranging from 1 (strongly disagree) to 4 (strongly agree). Higher values indicate higher levels of self-esteem. The respondents’ scores on each item were summed and divided by 4 to obtain an RSES score of 1 to 4. The questionnaire is valid and reliable, and the Norwegian version of the scale showed good psychometric properties [49]. Cronbach’s α in this study was 0.73.

*Loneliness* was measured using the revised UCLA Loneliness Scale (ULS-8) [50], which is a short version of the widely used 20-item revised ULS-20 [51]. ULS-8 consists of eight questions rated on a 4-point Likert scale with values ranging from “never” to “always”. The total score ranges from 8 to 32 points. Higher scores indicate a higher degree of loneliness [51]. The ULS-8 questionnaire was translated into Norwegian using standardized translation procedures and validated as part of the Start Young study [52]. Cronbach’s α in this study was 0.87.

*Stress* was measured using the 30-item Perceived Stress Questionnaire (PSQ) [53–55], which refers to the past four weeks and is answered using a 4-point rating scale ranging from 1 (rarely) to 4 (almost always). We recorded answers so that higher values indicate higher levels of perceived stress. The resulting PSQ total score was linearly transformed to a number between 0 and 1 using the equation PSQ = (raw value– 30)/90 [53]. The Norwegian version of PSQ has been shown to have good validity and reliability [55]. Cronbach’s α in this study was 0.87.

*Pain* was measured using selected questions from the Brief Pain Inventory (BPI) [56] and a few questions from the Lübeck Pain-Screening Questionnaire (LPQ) [57]. The BPI measures the subjective intensity of pain and how pain interferes with different aspects of life, and it has well-established validity and reliability internationally and in Norway [56, 58]. Pain interference questions were completed by those who scored ≥1 on the ‘pain on average’ question (indicating that they had pain) [58]. Respondents who rated ≥1 on this question of the BPI were given two follow-up questions from the LPQ about pain duration and pain frequency. The LPQ is a structured self-report questionnaire that is used to estimate the prevalence and consequences of pain [57]. The Norwegian LPQ has satisfactory feasibility, content and face validity [59]. Finally, two questions derived from the Norwegian “Pain, Youth and Self-Medication study” [60, 61] were used to measure the intake of pain analgesics. Respondents were asked about pain analgesic intake the past 4 weeks. If they answered “yes,” they were asked about the frequency of intake.

*HL* was measured using the Health Literacy Questionnaire (HLQ), which is a generic, multidimensional instrument [62] consisting of 44 items representing nine independent HL domains: (1) Feeling understood and supported by health-care providers (4 items); (2) Having sufficient information to manage my health (4 items); (3) Actively managing my health (4 items); (4) Social support for health (5 items); (5) Appraisal of health information (5 items); (6) Ability to actively engage with health-care providers (5 items); (7) Navigating the health-care system (6 items); (8) Ability to find good health information (5 items); and (9) Understanding health information (5 items). Domain numbers 1–5 are scored using response options ranging from 1 (strongly disagree) to 4 (strongly agree). Domain numbers 6–9 are scored using response options ranging from 1 (cannot do or usually difficult) to 5 (very easy). The domain scores were calculated as the average of the item scores. Higher scores indicate better HL. The Norwegian HLQ is considered valid and reliable [63]. Cronbach’s α in this study was ≥ 0.75 for all nine domains. Descriptive results are presented for the nine domains, however we only used the HLQ-domains numbers 2 and 8 in the multiple linear regression analyses because they were considered the most relevant for our purpose.

All questionnaires used in this paper had previously been translated into Norwegian. Most questions included a neutral option, resulting in all items being answered. All questionnaires that used sum scales showed satisfactory Cronbach’s alpha values above 0.7.

## Statistical analyses

### Sample size considerations

This is an association study, thus sample size considerations were related to precision of our estimates (width of confidence intervals) and number of covariates we were able to include in our multivariate models without overfitting. For multiple linear regression models, it is recommended to include 10–15 individuals per analysed covariate. Given we had 320 included individuals in our data set, we had enough statistical power to fit models with 21–30 covariates. As our models included nine covariates, we consider our study sufficiently powered.

All statistical analyses were conducted using IBM SPSS Statistics for Windows, Version 29.0 (IBM Corp., Armonk, NY, USA) and Stata, version 17. Demographic variables were described using descriptive statistics; continuous variables were described using mean, standard deviation (SD), and categorical variables with counts and percentages.

Bivariate analyses were performed using Chi-square test (pairs of categorical variables) or independent samples t test (continuous variables). Multiple linear regression analysis examined possible associations between demographic and psychosocial variables, gender, pain, HL, and HRQOL (RAND-36 PCS and RAND-36 MCS). The independent variables in the multiple regression analyses were the demographic variables of age, gender, marital status (cohabiting/living alone), education, pain, HL, self-efficacy, stress and loneliness. The selection of these variables was based on the fact that these variables were significantly associated with HRQOL in previous studies [12]. The level of potential multicollinearity between the independent variables was evaluated and was considered satisfactory. The level of significance was set at 0.05. All analyses are considered exploratory so no correction for multiple testing was performed.

## Results

In the present study, the mean age of the parents was 47.6 (SD = 4.6) years; 81% were mothers, 79% were married or cohabiting, 81% had a university degree, the majority worked full time (78%) or part time (13%), and 59% had a household income of more than 1 million Norwegian kroner (NOK). More mothers had part-time work and reported a household income of less than 1 million NOK compared with fathers (see Table 1). The average pain score was rather low, 0.48 (95% CI [0.43–0.54]). However, 50% of the parents reported persistent pain for more than three months. Compared with fathers, mothers reported a significantly higher average pain score of 0.51 (95% CI [0.45–0.57]) vs. 0.37 (95% CI [0.25–0.49])., p<0.001, pain interference, activity 1.58 (95% CI [1.27–1.89]) vs. 0.74 (95% CI [0.39–1.10]) vs., p<0.001, pain interference, emotions 1.9 (95% CI [1.60–2.27] vs. 1.09 (95% CI [0.66–1.52]) ), p<0.001, and more mothers reported pain for more than three months (53% vs. 37% vs., p<0.001) than fathers (see Table 2).

**Table 1:** Characteristics of the sample (*N* = 320) in 2021, including 258 mothers and 62 fathers

Demographics				*p* value
	All	Mothers	Fathers	
Age, years mean (SD)	47.6 (4.6)	47.3 (4.5)	49.1 (4.8)	0.427
*Marital status*				0.234
Married/cohabitating	252 (79%)	205 (79%)	47 (76%)	
Single	17 (5%)	16 (6%)	1 (1.5%)	
Divorced or separated	48 (15%)	35 (14%)	13 (21%)	
Widowed	3 (1%)	2 (1%)	1 (1.5%)	
*Education*				0.558
Compulsory education, post-compulsory, and certificate of apprenticeship	16 (19%)	52 (20%)	9 (14%)	
University <4 years	73 (23%)	57 (22%)	16 (26%)	
University ≥4 years	186 (58%)	141 (58%)	37 (60%)	
*Employment status*				0.001
Full time	250 (78%)	191 (74%)	59 (95%)	
Part time	42 (13%)	41 (16%)	1 (2%)	
Not working	28 (9%)	26 (10%)	2 (3%)	
*Absence from work in the last 3 months*				0.035
None	242 (76%)	186 (72%)	56 (90%)	
1–4 days	40 (12%)	35 (14%)	5 (8%)	
5–7 days	10 (3%)	10 (3%)	0	
8–10 days	3 (1%)	3 (1%)	0	
˃10 days	25 (8%)	24 (9%)	1 (2%)	
*Annual Household income (NOK)*				0.006
<450,000	19 (6%)	18 (7%)	1 (2%)	
451,000–750,000	50 (15%)	42 (16%)	8 (13%)	
751,000–1,000,000	163 (20%)	58 (23%)	5 (8%)	
˃1,000,000	188 (59%)	140 (54%)	48 (77%)	

Categorical data are presented as numbers (%) and continuous variables as mean (SD)

Chi-square tests and independent sample t tests were used to compare differences in categorical variables and continuous data, respectively

**Table 2:** Description of pain in the entire sample (*N* = 320) and separately for mothers (*N* = 258) and fathers (*N* = 62)

	All*	Mothers	Fathers	*p* value
Average pain score ^a^	0.48 (0.50)	0.51 (0.50)	0.37 (0.49)	<0.001
Pain interference, activity ^b^	1.43 (2.02)	1.58 (2.14)	0.74 (1.07)	<0.001
Pain interference, emotions ^b^	1.80 (2.17)	1.94 (2.26)	1.09 (1.31)	<0.001
Pain duration				0.075
No pain	96 (30%)	72 (28%)	24 (39%)	
≤3 months	64 (20%)	49 (19%)	15 (24%)	
˃3 months	160 (50%)	137 (53%)	23 (37%)	
Pain analgesics in the past 4 weeks				0.016
Yes	178 (56%)	152 (59%)	26 (42%)	
No	142 (44%)	106 (41%)	36 (58%)	
Frequency of pain analgesics in the past 4 weeks				0.635
Daily	23 (13%)	18 (12%)	5 (19%)	
Every week, but not daily	37 (21%)	33 (22%)	4 (5%)	
Less often than every week	116 (65%)	99 (65%)	17 (65%)	
No intake	2 (1%)	2 (1%)	0	

Categorical data are presented as numbers (%) and continuous variables as mean (SD).

Chi-square tests and independent sample t tests are used to compare differences in categorical variables and continuous data, respectively

^a^ Range: 0–10, where 10 indicates pain as bad as can be imagined

^b^ Range 0–10, where 10 indicates complete interference of pain

*Some parents do not respond to all questions, and thereby different number (n) for some variables

### HRQOL, psychosocial factors, and HL

The parents’ mean (SD) score for RAND-36 was 52.1 (95% CI [51.2–53.0]) for PCS and 51.0 (95% CI [50.0–52.1]) for MCS. Mothers reported significantly lower scores for all RAND-36 domains, including the PCS and MCS scores (Table 3). There were no gender differences regarding general self-efficacy, loneliness, stress, and self-esteem (Table 3).

**Table 3:** Descriptive characteristics of HRQOL, self-efficacy, self-esteem, loneliness, and stress (*N* = 320), and differences between mothers (*N* = 258) and fathers (*N* = 62)

	All	Mothers	Fathers	*p* value
*HRQOL*				
RAND-36 PCS^a^	52.1 (8.5)	51.6 (9.1)	54.1 (5.6)	<0.001
RAND-36 MCS^a^	51.0 (9.6)	50.4 (10.1)	53.7 (6.6)	<0.001
RAND-36 eight domains				
Bodily pain	78.2 (22.8)	76.6 (34.0)	84.4 (15.7)	<0.001
General health	76.1 (19.7)	75.0 (20.6)	80.5 (14.4)	0.016
Physical function	93.4 (13.8)	92.6 (14.9)	96.6 (7.1)	0.003
Physical role function	84.1 (31.5)	82.0 (33.1)	92.7 (21.9)	<0.001
Mental health	79.7 (14.0)	79.0 (14.7)	82.5 (10.2)	0.014
Vitality	63.2 (20.4)	61.7 (21.1)	69.4 (16.0)	0.017
Social function	86.8 (20.1)	85.4 (21.3)	92.7 (12.7)	<0.001
Emotional role function	83.0 (33.8)	80.5 (35.9)	93.5 (18.9)	<0.001
*Psychological factors*				
General self-efficacy ^b^	3.3 (0.4)	3.3 (0.4)	3.3 (0.4)	0.979
Loneliness ^c^	13.7 (4.2)	13.7 (4.2)	13.6 (4.2)	0.545
Stress ^d^	0.27 (0.16)	0.28 (0.16)	0.23 (0.14)	0.065
Self-esteem ^e^	3.3 (0.6)	3.3 (0.6)	3.5 (0.5)	0.112

Independent sample t tests were used to compare mothers and fathers

^a^ The score for the SF-36 ranges from 0 to 100, where 100 indicates a high HRQOL. PCS, physical component summary; MCS, mental component summary

^b^ Self-efficacy: range 1–4, where higher values indicate higher levels of self-efficacy

^c^ Loneliness: range 8–32, where higher values indicate higher levels of loneliness

^d^ Stress: range 0–1, where higher values indicate higher levels of stress

^e^ Self-esteem: range 1–4, where higher values indicate higher levels of self-esteem

In general, the parents reported favourable mean (SD) scores for the nine HL domains (Table 4), with the highest score, 4.1 (0.5) for the domain “Understand health information well enough to know what to do”. All the scores were higher than for the general Norwegian population. There were no statistically significant gender differences, considering the parents’ HL.

**Table 4:** Health literacy (HL) in 320 parents (258 mothers and 62 fathers)

	All *N* = 320	Mothers *N* = 258	Fathers *N* = 62	*p* value
HLQ_ Feeling understood and supported by health-care providers	3.12 (0.58)	3.14 (0.58)	3.06 (0.58)	0.797
HLQ_ Having sufficient information to manage my health	3.24 (0.47)	3.24 (0.47)	3.28 (0.47)	0.593
HLQ_ Actively managing my health	3.00 (0.53)	3.02 (0.54)	2.90 (0.51)	0.806
HLQ_ Social support for health	3.12 (0.53)	3.12 (0.54)	3.08 (0.48)	0.398
HLQ_ Appraisal of health information	2.86 (0.51)	2.86 (9.52)	2.85 (0.45)	0.362
HLQ_ Ability to actively engage with health-care providers	3.97 (0.58)	3.92 (0.59)	4.13 (0.52)	0.816
HLQ_ Navigating the health-care system	3.88 (0.58)	3.85 (0.58)	4.00 (0.57)	0.236
HLQ_ Ability to find good health information	3.99 (0.55)	3.97 (0.50)	4.07 (0.50)	0.993
HLQ_ Understand health information	4.08 (0.47)	4.07 (0.46)	4.14 (0.48)	0.520

Independent sample t tests were used to compare mothers and fathers

The range of the HL domains varies

### Associations between demographic variables, stress, psychosocial variables, HL, pain, and HRQOL

When applying multiple regression analysis for associations between demographic variables, stress, pain, psychosocial variables, HL, and HRQOL, we identified no associations between stress and RAND-36-PCS. However, a university education of four years or more was positively associated with RAND-36-PCS (B = 3.29, 95% CI [0.78–5.80]), while those who reported having pain for more than three months had on average 7 points lower PCS compared to those who did not report such pain (B= − 7.13,95% CI: [–9.20– − 5.06]).

In the multivariate model with RAND − 36 -MCS as the dependent variable, stress and age remained independently associated with this outcome. Parents who reported being stressed scored on average 43 points lower compared to parents who were not stressed (B = 43.11, 95% CI: [–48.83– − 37.38]). The older the parents were, the higher they scored MCS (B = 0.21, 95% CI: [0.04–0.39]) (Table 5).

**Table 5:** Associations between demographic and psychosocial variables, pain, and health literacy and HRQOL or HRQOL (RAND-36 PCS and RAND-36 MCS) examined by linear regression analyses (*N* = 320)

		RAND-36 PCS^e^			RAND-36 MCS	
	B	(CI)	*p* value	B	(CI)	*p* value
Gender (Ref = father)	–1.32	–3.58, 0.93	0.247	–1.12	–3.01, 0.76	0.243
Age	0.05	–0.15, 0.26	0.610	0.21	0.04, 0.39	**0.013**
Living conditions						
Married/cohabitating	ref			ref		
Single/divorced, widow/widower	0.86	–1.34, 3.08	0.441	–1.21	–3.05, 0.63	0.196
Education						
Less than 13 years of education	ref			ref		
University less than 4 years	1.42	–1.34, 4.18	0.312	–0.51	–2.81, 1.80	0.665
University 4 years or more	3.29	0.78, 5.80	**0.010**	0.28	–1.81, 2.38	0.789
Pain						
No pain	ref			ref		
Less than 3 months	–2.49	–5.00, 0.001	0.051	–0.41	–2.51, 1.68	0.697
More than 3 months	–7.13	–9.20, − 5.06	**<0.001**	0.13	–1.60, 1.86	0.880
Health literacy						
HLQ sufficient information	0.58	–1.78, 2.91	0.630	–0.20	–2.17, 1.76	0.838
HLQ ability to find information	0.94	–1.11, 2.98	0.367	1.07	–0.68, 2.78	0.219
Self-efficacy ^a^	–0.18	–2.56, 2.22	0.885	–0.44	–2.44, 1.56	0.663
Loneliness ^b^	–0.15	–0.39, 0.10	0.239	–0.12	–0.32, 0.08	0.236
Stress ^c^	–4.28	–10.81, 2.24	0.197	–43.11	–48.83, − 37.38	**<0.001**
R^2^ adj	22.2%			57.4%		

^a^ Self-efficacy: range 1–4, where higher values indicate higher levels of self-efficacy

^b^ Loneliness: range 8–32, where higher values indicate higher levels of loneliness

^c^ Stress: range 0–1, where higher values indicate higher levels of stress

^e^ The score for the SF-36 ranges from 0 to 100, where 100 indicates a high HRQOL. PCS, physical component summary; MCS, mental component summary

## Discussion

When exploring possible gender differences in stress psychosocial factors (self-efficacy, self-esteem, loneliness ), HL, pain and HRQOL in parents of adolescents one year into the COVID-19 pandemic, and the possible associations between gender, demographic and psychosocial variables, HL, and HRQOL, we made the following major observations. Mothers reported more pain than the fathers. Mothers reported lower HRQOL than the fathers. A university education of four years or more was positively associated with physical HRQOL, while persistent pain was negatively associated. Finally, stress was strongly negatively associated with mental HRQOL, while old age was positively associated. Resilience factors like self-efficacy and self-esteem were not associated with HRQOL.

In our study, mothers reported more pain than the fathers. This finding aligns with a systematic review based on various population-based studies that suggest women are more likely to experience a variety of chronic pain syndromes and tend to report more severe pain at a higher frequency and in a greater number of body regions than men [64]. Norwegian women generally report clinical pain more frequently, with longer duration and greater severity, than Norwegian men [65, 66].

Gender differences in the experience of pain may arise from different reasons. Psychosocial factors contribute substantially to human pain perception and may differentially influence pain in men and women [64]. One suggestion is that the gender difference could be caused by differences in the experience and processing of emotions that, in turn, differentially alter pain processing [67]. Another suggestion is that there are gender differences in communication and expression of pain [68]. Importantly, pain is a potential stressor, and a maladaptive perception of pain as threatening or frightening may evoke an exaggerated physiological stress response, thereby perpetuating chronic pain and disability [69, 70]. Several studies ranging from everyday hassles to post-traumatic stress reactions concluded that women subjectively experience more stress than men and frequently report more physical and somatoform symptoms, such as pain, and show higher stress vulnerability [70]. A stressor may be a physical or psychological threat to safety, status, or well-being; physical or psychological demands that exceed available resources; an unpredictable environmental change; or an inconsistency between expectations and outcomes [70]. The COVID-19 pandemic might be considered a stressor to some participating mothers.

Furthermore, mothers reported lower HRQOL than fathers in our study, which is not consistent with the recent Norwegian National Survey of QOL results that showed no differences in reported QOL between women and men [71]. However, in the national survey, QOL is measured with an overall question of satisfaction with life, and the results differ from the results of a measure of HRQOL. Moreover, gender differences in HRQOL have been reported in several studies across different age groups and health conditions [72]. The differences are most evident in the younger age group, where women generally report lower HRQOL than men. In a Swiss study, men reported better mental HRQOL (MCS) than women, but the difference was not evident in the physical HRQOL (PCS) [73]. Furthermore, mental HRQOL increased with age, while physical HRQOL deteriorated with increasing age. Interestingly, mental HRQOL was higher when living with a partner without responsibility for children, which aligns with findings from the Norwegian national survey [71, 73]. The findings from the Swiss [73] study is only partly consistent with our findings that gender differences are significant in both mental and physical HRQOL. The selected group might explain the differences in the current study, where all participants are parents of adolescents, and the stress of parenting combined with living through the COVID-19 pandemic can be explanatory factors.

Our results also show that experiencing persistent pain is associated with lower HRQOL in the PCS domain [74]. Earlier studies have shown that pain affects most domains of QOL, primarily physical and emotional functioning [74]. In the Norwegian national survey, those who report having medium or strong pain or discomfort reported significantly lower QOL than the population., Furthermore, our results show that stress was negatively associated with HRQOL. Stress is assumed to increase when higher demands and more insecurity are caused by the pandemic, such as medical conditions, fear of COVID, and worries about the children. Research has shown that parents have reported increased parenting stress during COVID-19 [75].

Both mothers and fathers in the present study reported higher scores on HL than the general Norwegian population [76] in the initial validation testing of the HLQ among nursing students and other adult people from the Western part of Norway in 2020. This was the case for all the mean scores of the nine HL domains [76]. Our sample of parents comprised well-educated mothers and fathers. In previous studies, high education and socio-economic status have been identified to be associated with higher HL [77, 78]. Although HL is not associated with HRQOL in the multiple analysis, high HL during the COVID-19 pandemic might be important to manage the crisis well.

### Strengths and limitations

A strength of the study is the many psychosocial variables included most of which are potential predictors of HRQOL in the group. The strength is supported by the explained variances of HRQOL of 22.2% for PCS and 57.4% for MCS in the final multivariate models. Another strength is that all variables were assessed using validated questionnaires and measures with favourable Cronbach α values [79].

One limitation of this study is the cross-sectional nature, which reveals only statistically significant associations between the variables and does not allow one to conclude causality. Furthermore, the characteristics of the parents, which included mainly mothers, married/cohabiting, and well-educated adults with a high household income (the median household net income for couples with children/adolescents was about 950,000 NOK/year [80]), limit the ability to generalize our findings to the entire population of Norwegian parents. Finally, the small number of parents from the lower socio-economic classes and the low overall response rate indicates that the study may have introduced selection bias because of the high proportion of parents who did not participate in the survey.

### Implications and future research

The study contributes to knowledge about how stress, psychosocial factors HL and pain are related to HRQOL in parents of adolescents with high socio-economic status during the COVID-19 pandemic. This knowledge may help to inform policymakers, politicians, and health-care professionals about prioritizing and guiding parents in stressful situations. The high proportion of parents reporting pain and the strong association between pain and HRQOL suggest that more attention should be paid to pain and pain management, including mental pain and the stressful situation in parents.

For future research, we suggest using longitudinal designs to explore our findings more thoroughly with data before, during, and after the COVID-19 pandemic. Future research should include parents with a more heterogeneous socio-economic background. Furthermore, studies should control for other possible confounders and add more health-related data (e.g., about exercise).

## Conclusion

One year into the COVID-19 pandemic, mothers reported more pain and lower HRQOL compared to fathers. During the COVID-19 pandemic, results indicate that mothers, parents with persistent pain and those experiencing stress might be prone to a decrease in their HRQOL. It is possible that relevant information and guidance adapted to parents of adolescents can prevent stress which in turn can positively affect HRQOL. Furthermore, we suggest designing HRQOL interventions targeting mothers, parents in pain and those with lower education.

## Data Availability

The datasets used and/or analyzed during the current study are not publicly available due to General Data Protection Regulation laws but are available from the corresponding author on reasonable request and with permission from the Norwegian Centre for Research Data.
